# An Exploration of Human Well-Being Bundles as Identifiers of Ecosystem Service Use Patterns

**DOI:** 10.1371/journal.pone.0163476

**Published:** 2016-10-03

**Authors:** Maike Hamann, Reinette Biggs, Belinda Reyers

**Affiliations:** 1 Stockholm Resilience Centre, Stockholm University, Stockholm, Sweden; 2 Centre for Complex Systems in Transition, Stellenbosch University, Stellenbosch, South Africa; 3 Natural Resources and Environment, Council for Scientific and Industrial Research, Stellenbosch, South Africa; University of Alabama, UNITED STATES

## Abstract

We take a social-ecological systems perspective to investigate the linkages between ecosystem services and human well-being in South Africa. A recent paper identified different types of social-ecological systems in the country, based on distinct bundles of ecosystem service use. These system types were found to represent increasingly weak direct feedbacks between nature and people, from rural “green-loop” communities to urban “red-loop” societies. Here we construct human well-being bundles and explore whether the well-being bundles can be used to identify the same social-ecological system types that were identified using bundles of ecosystem service use. Based on national census data, we found three distinct well-being bundle types that are mainly characterized by differences in income, unemployment and property ownership. The distribution of these well-being bundles approximates the distribution of ecosystem service use bundles to a substantial degree: High levels of income and education generally coincided with areas characterised by low levels of direct ecosystem service use (or red-loop systems), while the majority of low well-being areas coincided with medium and high levels of direct ecosystem service use (or transition and green-loop systems). However, our results indicate that transformations from green-loop to red-loop systems do not always entail an immediate improvement in well-being, which we suggest may be due to a time lag between changes in the different system components. Using human well-being bundles as an indicator of social-ecological dynamics may be useful in other contexts since it is based on socio-economic data commonly collected by governments, and provides important insights into the connections between ecosystem services and human well-being at policy-relevant sub-national scales.

## Introduction

Human well-being is dependent on ecosystems for provisioning and regulating services like food, clean drinking water, and protection from hazards such as floods, as well as cultural services such as spiritual enrichment and recreation [1, 2]. Global environmental change threatens the supply of essential ecosystem services, and it is imperative to understand how such changes may influence human well-being into the future [3–5]. Yet despite extensive research in recent years, many gaps in our knowledge of the linkages between ecosystem services and human well-being remain [6–8]. In part, this is due to a lack of studies that empirically measure both ecosystem services and human well-being, and that consider the social-ecological systems within which these interactions take place [7, 9].

An understanding of the type of social-ecological system that underpins the interactions between ecosystems and society can yield important insights into resource use patterns and dynamics. For example, in a recent review Cumming *et al*. [10] identified different types of social-ecological systems based on the strength of the connection between people and their local environment. In rural “green-loop” systems, where people depend on local ecosystems and the services they provide, feedbacks between ecosystems and societies are direct and clear. However, as populations grow, production roles become more specialized, market economies develop, and fewer people come into direct contact with local ecosystems. These societies transition to a “red-loop” system, in which resources are no longer extracted mainly from local ecosystems, but instead obtained from far-away places and impacts are outsourced. The result is a weakening of feedbacks between ecosystem services and society, so that people’s reliance on ecosystem services becomes obscured.

Applying this system typology, Hamann *et al*. [11] mapped social-ecological systems in South Africa, based on bundles of ecosystem service use. The bundles consisted of six locally-sourced provisioning services that cover people’s basic needs: animal production, crop production, freshwater from a natural source (river or spring), natural building materials, wood for cooking, and wood for heating. The level of direct use of these six services among households was determined from census data for each municipality, and subjected to a cluster analysis to identify three distinct types of ecosystem service use bundles across South Africa. The three bundle types represented an overall high, medium and low level of direct ecosystem service use among households, which was taken to correspond, broadly, to “green-loop”, “transition”, and “red-loop” systems, as defined by Cumming *et al*. [10].

Beyond differences in the level of use of or dependence on ecosystem services, social-ecological systems are characterized by other fundamental differences in structure and dynamics. Cumming *et al*.’s [10] system typology indicates that the green- to red-loop transition brings with it major shifts in the way society operates: As populations grow and become more urbanized and disconnected from their local ecosystems (while becoming increasingly dependent on natural resources sourced from far-away ecosystems), economies scale up, the work force becomes specialized and wide-spread infrastructure development takes place. Hospitals and universities are built, and technology advances. In these red-loop dynamics, traditional well-being measures like income and life expectancy tend to improve. In contrast, green-loop dynamics keep the community closely connected to their local ecosystems, but a lack of specialization and technological advancement means that these communities often do not engage in broader market-based economies, so that well-being measures such as income and health may not improve. We therefore expect that different social-ecological systems will not only express different ecosystem service use patterns, but also different human well-being patterns.

This paper aims to test this proposition by comparing the distribution of human well-being bundles and ecosystem service use bundles in South Africa. This approach is significant on two levels: Firstly, taking a bundles approach may help to disentangle some of the complexities inherent in the relationship between ecosystem services and human well-being. Using ecosystem service and human well-being bundles to identify different kinds of social-ecological systems in a landscape may tell us more about the area’s ecosystem services, human well-being, and linkages between the two than an analysis focusing on individual indicators. Secondly, data on a variety of human well-being and development indicators are often more widely available than data on ecosystem services and their use. If different social-ecological systems and their characteristic resource use dynamics can be identified using human well-being indicators, instead of ecosystem services, then it could assist with ecosystem management in data poor regions.

Our primary objective is therefore to ascertain to what extent distinct human well-being bundles correlate with ecosystem service use bundles, and whether human well-being bundles can be used as a proxy to identify the same social-ecological systems that were identified with the ecosystem service use bundles defined by Hamann *et al*. [11]. To achieve this objective we construct a multidimensional well-being bundle based on publicly available indicators that together represent the Millennium Ecosystem Assessment’s (MA) [1] five constituents of human well-being: security (e.g. personal safety, security from disasters); basic material for a good life (e.g. sufficient nutritious food, shelter); health (e.g. strength, access to clean air and water); good social relations (e.g. social cohesion, mutual respect, ability to help others); and freedom of choice and action. While human well-being is a composite measure that has been defined in many different ways [12, 13], we chose an indicator bundle that captures the MA’s well-being constituents because these constituents cover a broad and inclusive definition of human well-being and can be represented by indicators that are commonly collected by governments. In addition, even though their conceptual connection to ecosystem services was outlined in the MA, few empirical studies have explored these links at above-local scales. We also investigate the relationship between the ecosystem service and human well-being bundles, as well as their individual indicators, and find significant connections at a sub-national scale. In addition, we evaluate the bundles approach by analysing similarities and differences between the human well-being bundles we identified and more traditional well-being measures like economic activity and a South African deprivation index.

## Materials and Methods

We mapped human well-being in South Africa using a bundle of well-being indicators and compared the distribution of well-being bundles to that of ecosystem service use bundles, as well as other widely-used well-being indicators. Our unit of analysis was the municipality, which is the smallest administrative unit controlled by a local government council in South Africa. There are a total of 234 municipalities in the country, with an average size of 5217 km^2^ (range: 252–36 128 km^2^) and an average of number of 61 753 households (range: 1784–1 434 856).

### Selection of human well-being indicators for a well-being bundle

South Africa is a market-based economy, the 2^nd^ largest in Africa after Nigeria, and the dominance of cash-based exchanges reaches into the most rural areas, supported by a large-scale social grant system [14]. Aspirations for modern lifestyles and economic opportunities are evidenced by the high migration rates to urban areas, especially among the young [15, 16]. This context played an important role in selecting indicators that reflect current South African realities in terms of human well-being.

The five constituents of human well-being identified by the MA [1]–i.e. basic material for a good life, health, security, good social relations, and freedom of choice and action—were used as a guide in the choice of indicators for a human well-being bundle (Table 1). All data were obtained from the most recent South African population census conducted in 2011 [17], and can be freely accessed via www.statssa.gov.za. The indicators were selected based on their ability to represent one or more of the MA’s well-being constituents. For example, average household income was selected as an indicator of having a livelihood and being able to afford the materials to cover one’s basic needs (such as food, shelter and clothes). However, income may also contribute to health by providing access to healthcare. Furthermore, having an income provides opportunities that support freedom of choice and action. Average age of death is another indicator of health, and was chosen because life expectancy at birth, a more common health indicator, is not available for South Africa at the municipal level. Property ownership can be considered an indicator of security, since owning property indicates secure tenure and assets. In South Africa, property ownership is not necessarily tied to income, since traditional tenure systems and government housing schemes allow property ownership even when households are poor. Employment is a well-being indicator for a number of different well-being constituents, including basic material for a good life, and security. In addition, the percentage of people who are unemployed or discouraged work-seekers can be considered an indicator for the status of social relations in the municipality, especially in the context of a predominantly cash-based economy like South Africa. The MA characterized “good social relations” by social cohesion, mutual respect and the ability to help others. Being unemployed or even discouraged in one’s efforts to find a job negatively impacts social relations, since having a job not only contributes to income but also conveys status in the community, elicits respect by others, and fosters self-esteem [18, 19]. At the aggregate level (e.g. municipalities or nations), unemployment may lead to increased inequality and social tension, and has been linked to increased crime rates [20–22]. While all the indicators described so far also contribute to freedom of choice and action, education is an especially important indicator for this well-being constituent, as a good education leads to employment opportunities and allows people to realize their life goals and participate more fully in society. Higher education levels within a population are also strongly correlated with civic engagement and democracy [23], which enhance personal freedom of choice and action. A correlation analysis of the indicators making up the well-being bundle was performed and visualized using the *corrplot* function in R [24] (S1 Fig).

**Table 1:** Selected indicators for a human well-being bundle capturing the five well-being constituents of the Millennium Ecosystem Assessment (MA) [1]: Basic material for a good life; health; security; good social relations; freedom of choice and action. All data were sourced from the South African National Census 2011 [17].

Indicator	Measure (per municipality)
Income	Average annual household income (including grant incomes)
Life span	Average age of death
Property ownership	Percentage of households where dwelling is owned and fully paid off
Unemployment	Percentage of people who are unemployed or discouraged work-seekers
Education	Percentage of people who have completed secondary schooling or have some form of tertiary education

Our indicator selection is subject to data availability at a national scale and we acknowledge that our choice represents one of many possible human well-being bundles, but we feel that the bundle chosen here reasonably captures the well-being dimensions identified by the MA relevant to the South African context. To assess the sensitivity of our results to indicator selection, we repeated the analysis with a different “basic material for a good life” indicator, namely a poverty line measure. Average income may mask a skewed distribution of income within a municipality. This alternative indicator represented the level of poverty, measured in terms of the percentage of households per municipality that lived above a certain poverty threshold. This threshold was defined as an annual household income of more than ZAR 76 400, based on national poverty line data and census data income bands [17, 25].

### Mapping human well-being bundles

After calculating the values for each human well-being indicator per municipality, the data were visualized and analysed in ArcGIS 10.0 [26]. The spatial clustering of all services was determined using spatial autocorrelation (Global Moran’s I statistic [27]). To identify groups of municipalities that shared similar combinations of values for the five well-being indicators, the values for all indicators were first scaled between 0–1, and then a *k*-means cluster analysis was performed using the Hartigan–Wong algorithm [28] with 25 random starts and a maximum of 10000 iterations to find the cluster solution with the lowest within-cluster sum of squares. The analysis was performed in R using the *kmeans* function from the stats package [29]. *K*-means clustering (number of clusters = 3) was chosen to repeat the clustering procedure used by Hamann *et al*. [11]. Results of the cluster analysis are visualised in a multidimensional scaling ordination diagram based on the dissimilarities in the human well-being measures between all the municipalities [30]. Multidimensional scaling was performed and plotted in R using the *cmdscale* and *plot* functions from the stats and graphics packages [29]. Since the clustering method can have an effect on which clusters are found in the data, hierarchical clustering was also performed to compare results between the different clustering algorithms. In addition, cluster validation procedures using the *clValid* package in R [31] indicated support for two clusters within the data, which was likewise investigated for comparative purposes. Human well-being bundles were visualized using star plots in R and clusters were mapped in ArcGIS.

### Exploring the relationship between human well-being and ecosystem service use

The spatial distribution of human well-being bundles was compared to that of ecosystem service use bundles as determined by Hamann *et al*. [11]. The overlap between the different human well-being and ecosystem service bundle types was calculated in terms of area and population. To analyse relationships between individual indicators and bundle types, a multinomial logit model using the *mlogit* package in R [32] was run on the human well-being bundle types as the dependent variable and individual ecosystem service use indicators as predictor variables, as well as vice versa for ecosystem service bundles and individual well-being indicators (standardized to *z*-scores).

### Comparing human well-being bundles and other well-being indicators

To compare our human well-being bundle to more traditional measures of well-being like GDP and the Human Development Index (HDI), we calculated the Gross Value Added (GVA) for each municipality from the South African Geospatial Analysis Platform [33]. GDP figures are not publicly available at the municipal level, but GVA is related to GDP and is a measure of regional economic activity. As an example of a well-being index, we used the South African Index of Multiple Deprivation (SAIMD) which is a publicly available index of well-being at the municipal level that was derived for the national Department of Social Development from the nation-wide 2007 community survey [34, 35]. It is composed of a total of eleven indicators that reflect four dimensions of deprivation: income and material deprivation, employment deprivation, education deprivation, and living environment deprivation (the latter referring to poor living conditions). Even though the data used to compile the GVA figures and SAIMD ranks precede the census data by 2 to 4 years, we assume the patterns to not have changed significantly between data compilations. Differences in *per capita* GVA scores and SAIMD ranks between municipalities in different human well-being bundle types were visualized in R using boxplots. Data were tested for normality using the Shapiro-Wilk test [36]. Since the *per capita* GVA data were of unequal sample size, found to contain outliers and non-normally distributed, they were log-transformed before being subjected to Welch’s t-tests on unique pairs [37]. Differences in deprivation ranks between pairs of human well-being bundle types were assessed for significance using Mann-Whitney-Wilcoxon tests [38].

## Results

**Fig 1:**
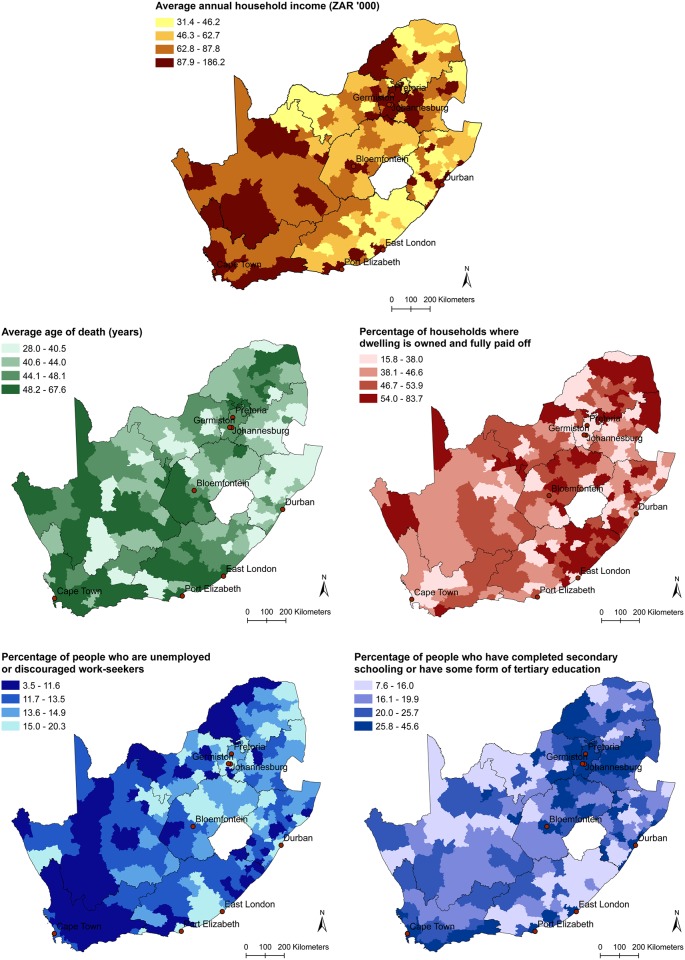
Human well-being indicators for South Africa. Values are categorized in quartiles. All indicators were found to be significantly clustered in space (Moran’s I, p < 0.01).

Mapping the five human well-being indicators showed that elevated levels of well-being were mostly found in the western and north-eastern parts of the country (Fig 1). The exception was property ownership, where high percentages of households that own their dwelling were found along the south-east coast where incomes are typically low.

### Human well-being bundles

**Fig 2:**
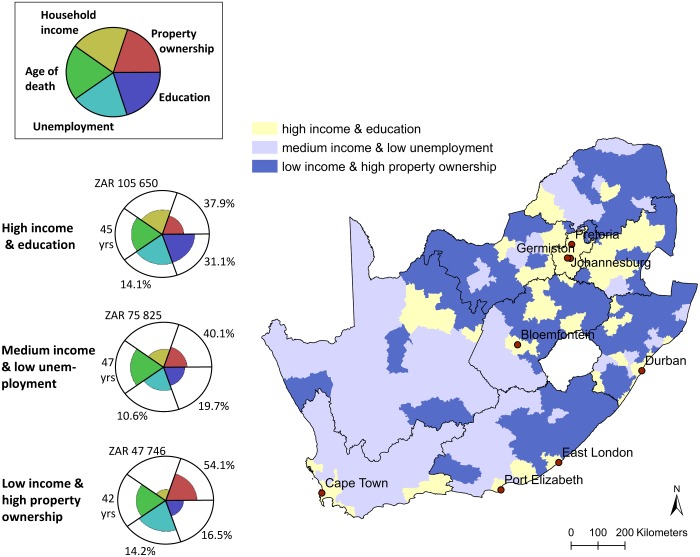
Human well-being bundle types representing three distinct configurations of human well-being in South Africa. Values are averages for each indicator (see Table 1 for an explanation of the units), calculated across all municipalities that were clustered together within a bundle type. Petal length represents the average value (relative to the absolute maximum) of each indicator among the municipalities that share a similar bundle type. The map shows the distribution of the three different bundle types in South Africa. Areas of the same colour share similar well-being bundles at the municipal level and are significantly clustered in space (Moran’s I, p < 0.01). Provincial borders and major metropolitan centres are shown.

A cluster analysis of the municipalities based on their values for the five human well-being indicators resulted in three distinct types of well-being bundles (Fig 2). The first bundle type exhibited high levels of income and education, but unemployment was high and property ownership was the lowest of all bundle types. The second bundle was characterised by medium income, the highest age of death and lowest unemployment rate. The third bundle type exhibited overall low levels of well-being compared to the other bundle types, with the highest unemployment, and the lowest household income, age of death, and education levels. However, the percentage of households in a municipality that own their dwelling and have paid it off fully was higher in this bundle than in the other two—due to historical and cultural tenure arrangements in South Africa (see discussion). In order to facilitate the discussion of results, these bundles are henceforth referred to as the “high income”, “medium income” and “low income” bundle, respectively. We use income to name the bundles, as it is one of the indicators that varied most clearly between the different bundle types, but emphasize that the bundles are multidimensional and represent far more than differences in income.

When the different well-being bundle types were mapped across South Africa (Fig 2), the resulting pattern of human well-being was significantly clustered in the landscape (Moran’s I, p < 0.01). In total, 60 of the 234 municipalities fell into the high income bundle, 63 municipalities fell into the medium income bundle, and 111 municipalities fell into the low income bundle. That corresponded to 15.3%, 46.2% and 38.5% of the total land surface area, and 60.7%, 7.0% and 32.3% of the total population, respectively. The high income bundle occurs mainly in densely populated regions around metropolitan centres, while the medium income bundle occurs in the sparsely populated and rural central and western regions of the country. The low income bundle occurs mostly in more densely populated rural and semi-rural areas in the east of the country.

A classical multidimensional scaling analysis of the municipalities and their human well-being measures revealed a clear delineation of the three bundle types identified in the cluster analysis (S2 Fig). When the clustering algorithm is restricted to two clusters, the medium income bundle area splits roughly equally into either high or low income bundle areas (S3 Fig). When hierarchical clustering is applied, the three-cluster pattern looks similar to that obtained after k-means clustering (S4 Fig), though more areas are classified within the low income bundle. If income is replaced with a measure of poverty to test the sensitivity of the analysis to indicator selection, 11% of the municipalities were assigned to a different bundle type than in the original analysis.

### Comparison of human well-being and ecosystem service bundles

**Fig 3:**
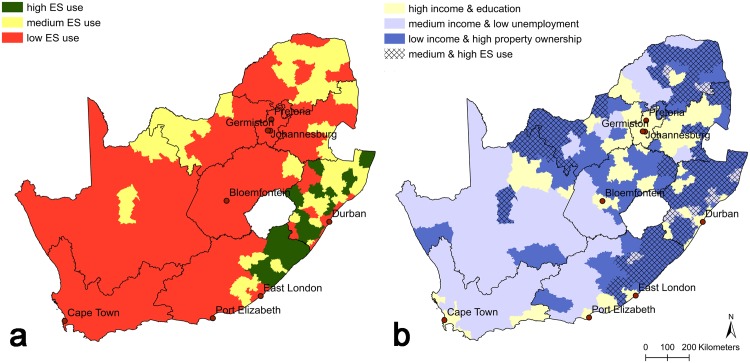
Distribution of a) ecosystem service (ES) use bundles in South Africa, as defined by *Hamann et al*. [11]; and b) the overlap between ES use and human well-being bundles. The cross-hatched areas in (b) represent systems characterized by high and medium ES use among households.

We found that the distribution pattern of human well-being bundles mirrored, to a substantial extent, the pattern in ecosystem service use bundles as mapped by Hamann *et al*. [11] (Fig 3): 93% of high and medium direct ecosystem service use areas coincided with the low income bundle areas.

**Fig 4:**
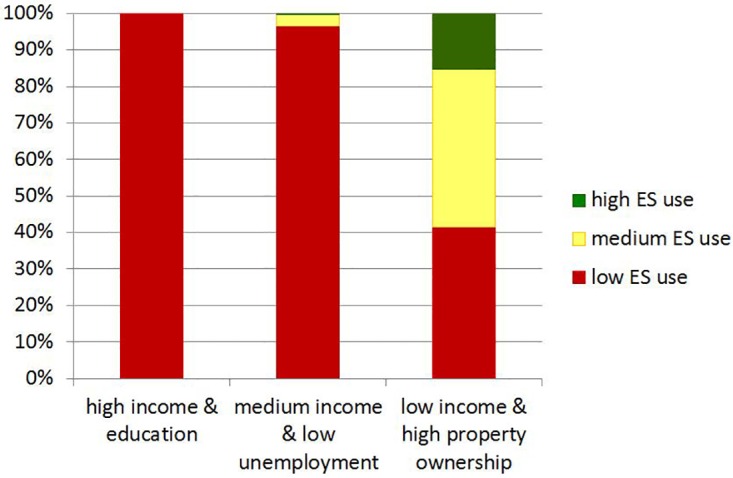
Percentage overlaps in area between different ecosystem service (ES) use and human well-being bundles. The different ES use areas make up a percentage of the total area of each well-being bundle.

The relative area comparison between the two maps shows that there was no overlap between high or medium ecosystem service use areas and high income bundle areas (Fig 4). Only a very small percentage (3.5%) of the area characterized by the medium income bundle also exhibited high or medium levels of ecosystem service use. In low income bundle areas, low and medium levels of ecosystem service use were found in nearly equal proportions (41.4% and 43.5%, respectively), while 15.2% of the area exhibited high levels of ecosystem service use.

When considering the percentage of *total* land area and population in South Africa (Table 2)–as opposed to the *relative* area of different ecosystem service use and well-being areas (Fig 4)–the combination of low levels of ecosystem service use and the well-being bundle characterized by high income and education covered 60.7% of the total population, but only 15.3% of the total area. In contrast, while almost half the country’s area was covered by the combination of low ecosystem service use and the well-being bundle characterized by medium income and low unemployment, it translates to only 5.5% of the population (Table 2).

**Table 2:** Overlaps between human well-being and ecosystem service (ES) use as percentages of South Africa’s total land area and population.

Human well-being	ES use	Area (%)	Population (%)
low income & high property ownership	low	15.9	8.1
	medium	16.7	14.9
	high	5.8	9.2
medium income & low unemployment	low	44.6	5.5
	medium	1.5	1.5
	high	0.1	0.1
high income & education	low	15.3	60.7
	medium	0	0
	high	0	0
Total		100	100

### Relationships between single indicators and bundle types

In terms of determinants of bundle membership, we found that an increase in average household income and age of death significantly increases the log-odds of a municipality being classified as part of the medium or low ecosystem service use bundle (Table 3). The other well-being indicators do not play a significant role in determining the bundle of ecosystem service use in South African municipalities. The results also show that the human well-being bundle type is sensitive to changes in the level of animal and crop production. Similarly, an increase of the percentage of households that make use of natural sources like rivers or springs for freshwater or that use wood for heating significantly decreases the log-odds of a municipality being classified as part of the high income and education well-being bundle.

**Table 3:** Estimated model coefficients and their standard errors (in parentheses) for changes in the classification of municipalities into direct ecosystem service (ES) use or human well-being bundles, in response to individual well-being and ES use indicators.

	**Direct ES use bundle**	
	**high → med**	**high → low**
**Household income**	5.787 (1.943)**	8.939 (2.034)**
**Property ownership**	0.404 (0.313)	0.608 (0.367)
**Education**	1.329 (0.901)	1.872 (0.985)
**Unemployment**	-0.172 (0.509)	-0.242 (0.55)
**Age of death**	1.23 (0.504)*	2.145 (0.551)**
	**Human well-being bundle**	
	**low → med**	**low → high**
**Animal production**	-0.137 (0.033)**	-0.302 (0.069)**
**Crop production**	-0.126 (0.041)**	-0.091 (0.05)
**Freshwater**	0.042 (0.034)	-0.281 (0.139)*
**Building materials**	0.012 (0.027)	0.233 (0.074)**
**Wood for cooking**	-0.037 (0.026)	0.072 (0.054)
**Wood for heating**	0.052 (0.034)	-0.182 (0.064)**

Human well-being bundles that are referred to as “high”, “med”, and “low” correspond to the “high income and education”, “medium income and low unemployment” and “low income and high property ownership” bundles, respectively. Significance at p < 0.05 denoted by * and at p < 0.01 by **.

### Comparison of human well-being bundles and other well-being indicators

**Fig 5:**
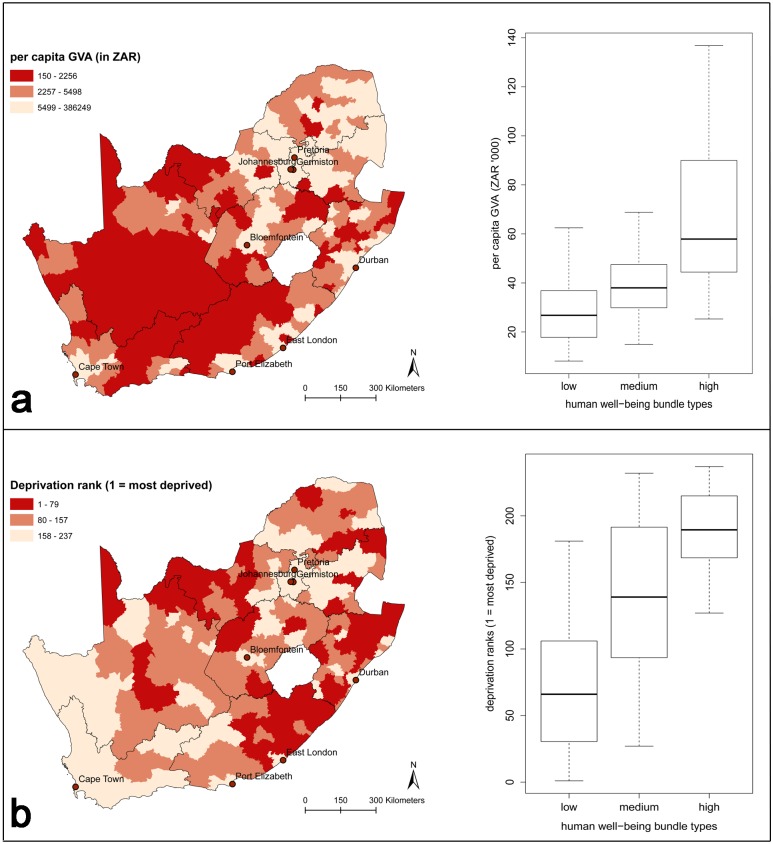
a) *Per capita* Gross Value Added (GVA) in South African Rand (ZAR), and b) the South African Index of Multiple Deprivation (SAIMD), per municipality. Values are categorized in quantiles; provincial borders are shown. The boxplots on the right of the maps show the difference in *per capita* GVA (in ZAR ‘000) and in SAIMD ranks (1 being the most deprived) between the different human well-being bundle types. Human well-being bundle labels are abbreviated to “high”, “med”, and “low”, corresponding to the high, medium and low income bundles. Outliers in the *per capita* GVA data were excluded from the boxplot.

When comparing *per capita* GVA and SAIMD ranks across the human well-being bundle types (Fig 5), the *per capita* GVA scores were significantly different between all pairs of bundles: the high (mean_high_ = 4.83, SD = 0.26) and medium income (mean_medium_ = 4.61, SD = 0.22, t (117) = 5.14, p < 0.001), high and low income (mean_low_ = 4.43, SD = 0.25, t (120) = 9.75, p < 0.001), as well as the medium and low income bundles (t (144) = 4.80, p < 0.001). *Per capita* GVA was highest in the high income and education bundle, and lowest in the low income and high property ownership bundle. Wilcoxon rank sum confirmed significant differences between all pairs of bundle types for the deprivation ranks of municipalities (the lower the rank, the more deprived the municipality). The median rank of the municipalities in the high income bundle (median_high_ = 189.5) differed significantly from that of the medium income bundle (median_medium_ = 139, U = 2919, n_1_ = 60, n_2_ = 63, p < 0.001). The same was found for the high and low income bundles (median_low_ = 66, U = 6559, n_1_ = 60, n_2_ = 111, p < 0.001), and for the medium and low income bundles (U = 1390, n_1_ = 63, n_2_ = 111, p < 0.001). The level of deprivation among municipalities therefore increased from the high, to medium, to low income bundles.

## Discussion

This study used a bundles approach to map human well-being at a sub-national scale, and investigates whether different human well-being bundles and ecosystem service use bundles show similar distributions across South Africa. We found three distinct human well-being bundles which reflected differing levels of multidimensional well-being across the country. These human well-being bundles were found to mirror the distribution of ecosystem service use bundles to a substantial degree, suggesting that well-being bundles could be used as proxies for ecosystem service use and underlying social-ecological dynamics in data poor areas. Our results show that well-being across South Africa is differentiated and nuanced, and may not be adequately reflected by single well-being indicators or composite indices that mask diversity between well-being constituents.

### Well-being bundles and overlap with ecosystem service use bundles

The main distinction between the medium or high income bundle and low income bundle was property ownership. The high levels of property ownership in the otherwise low well-being bundle may be explained by the high proportion (26.4%) of land under the rule of traditional authorities that makes up the low income bundle areas in South Africa. In these communal lands a property may be acquired from a local traditional leader or village chairman in charge of land allocations, usually for a nominal fee [39], which means that people with otherwise few assets have access to property without the usual risks incurred by lower-income homeowners in other countries (like low rates of appreciation and subprime mortgages) [40, 41]. Globally, land titles are argued to convey social and economic empowerment to their owners, particularly if they are poor, as the titles represent assets that can be used as collateral security and thereby promote upward mobility [42, 43]. However, it is debated whether this holds true in the global South, and especially in South Africa [44–47]. The title deeds held in communal areas do not necessarily promote lending to the poor, since banks are generally not willing to expose themselves to the high risk of non-repayment on land parcels with relatively low market value. However, while these homes may not lead to upliftment out of poverty in the form of income or credit, they nevertheless provide security of shelter (especially in old age) and represent a tie to one’s community that may contribute to well-being [48–50].

Our results support the fact that it is the poorer and often most vulnerable sectors of society who depend most on their immediate natural environment to meet their basic needs [1, 51, 52], but not in all cases. We find that high and medium ecosystem service use areas are found almost exclusively in low income bundle areas (Fig 3b), which constitute 24.1% of the population (Table 2), or 12.5 million people. Conversely, all of the high income bundle areas, as well as the vast majority of the medium income bundle areas, overlapped with the low ecosystem service use areas (Fig 4). However, a large proportion of the low income bundle area also overlaps with low levels of ecosystem service use. This most likely reflects the fact that in South Africa, many people may cover their basic needs with the help of social grants, which are distributed to almost 30% of the population [14, 53]. Low levels of human well-being across the majority of indicators chosen here therefore do not necessarily imply high levels of ecosystem service use or dependence, but high levels of ecosystem service use among households almost exclusively implies relatively low overall well-being.

### Identifying green-loop and red-loop systems with human well-being bundles

Hamann *et al*. [11] used bundles of ecosystem service use to identify green-loop, transition and red-loop systems in South Africa. These systems are theorized to represent distinct areas characterized not only by differences in the level of ecosystem service use among households, but also by differences in the broader socio-economic dynamics that affect human well-being [10]. We investigated whether well-being bundles would be able to act as proxies for ecosystem service use bundles in identifying these different system types. As outlined above, the distribution of human well-being bundles was only partly concordant with the distribution of low, medium and high ecosystem service use bundles—representing red-loop, transition and green-loop systems, respectively. While generally high levels of well-being (high and medium income bundles) are able to identify 79% of red-loop areas, low levels of well-being (low income bundles) were not able to distinguish between green-loop, transition or red-loop systems.

This lack of congruence in low income bundle areas could be due to multiple factors, including the particular well-being indicators used in this study. We also hypothesize that this discrepancy between ecosystem service use and low human well-being may be due to a difference in slow and fast-changing variables [54]. The level of direct use of locally-sourced ecosystem services may change within very short timeframes at the household level. When electrification, sanitation and infrastructural development of an area takes place, households may switch within months from using fire wood for cooking and fetching water from a stream to using electric ovens and the municipal water supply, i.e. the use of locally available natural resources diminishes rapidly. However, the indicators of well-being that were included in this study (especially education, average life span and unemployment) are slower to improve, which may explain the areas in which both human well-being and ecosystem service use are relatively low. Our results therefore suggest that transformations from the green-loop to the red-loop system do not always imply an immediate improvement in human well-being. Current well-being may reflect ecosystem service use levels from ten years ago, not present levels. It has been proposed that similar time lags contribute to the reverse situation, i.e. when use of ecosystem services is high and the environment is being degraded, yet human well-being does not diminish (termed the “environmentalist’s paradox”) [8].

Differences between slow and fast-changing variables, and specifically the temporal dynamics of human well-being compared to ecosystem service use patterns, raises important questions about attempts to investigate the linkages between ecosystem services and human well-being using data from a single point in time. Maps, especially, portray a snapshot in time, often with the implicit assumption that processes captured by the image are in some kind of equilibrium. If, as our results suggest, there is a delay in the human well-being variables when systems shift from green-loop to red-loop dynamics, then it is more appropriate to observe and measure ecosystem service use and human well-being patterns over an extended period of time. This has implications for research design in the field of ecosystem service science, and the importance of longitudinal or repeated cross-sectional data in untangling the links between ecosystem services and human well-being.

### Predictors of human well-being and ecosystem service use bundles

An increase in household income appears to play the dominant role in moving from high to low levels of ecosystem service use (Table 3). The only other significant predictor was average age of death. In our case, the overall level of ecosystem service use in a municipality therefore appears to relate most closely to well-being indicators representing the basic material for a good life, and health.

At the same time, as income and overall well-being increases, animal and crop production at the household level decreases (Table 3). As households transition from lower to higher income levels this may compromise food security and resilience to socio-economic upheaval. In South Africa—like in most developing countries—areas where well-being is generally low are mainly rural communities in which some proportion of household income is made up of remittances from household members working in urban centres as labour migrants [55–57]. Increases in household income through remittances may be relatively small compared to incomes in wealthy urban centres, but they may be large enough for rural households to transition into low ecosystem service use dynamics, abandoning local small-scale agriculture in the process, particularly if the remaining rural household members are elderly, sickly, or too young to farm [58, 59]. If, however, the remittances cease due to unforeseen circumstances (e.g. the wage earner’s death, illness, or job loss) the household is left with a much reduced income and no safety net in the form of small-scale subsistence farming [60].

### Comparison of human well-being bundles and other well-being indicators

Using bundle types to map human well-being allows a comparison of different constituents of well-being across an area. Such an analysis of differences between well-being components is often lacking in well-being analyses based on widely-used one-dimensional indicators like income or composite indices like the Human Development Index (HDI) [61], which tend to reduce the complexity and mask nuances [62–64]. In our case, average *per capita* GVA and South Africa’s deprivation index corresponded to the income component of our bundles (Fig 5). However, both the *per capita* GVA and deprivation index miss the covariation of well-being constituents across the landscape as identified by the bundles approach. For example, the medium income bundle areas exhibit lower levels of economic activity and have a lower annual household income than the high income bundle areas, but unemployment is lower and life spans are longer in these mostly rural areas. Overall well-being may therefore arguably be higher in the medium income bundle than in the high income bundle, even though the economic data would suggest otherwise. On the other hand, single indicators have the advantage of being transparent and easily communicated. Choosing well-being measures will therefore always be a subjective exercise and must be tailored to requirements [63].

### Limitations and further research needs

The bundles approach applied here contributes to a richer understanding of the pattern of multidimensional human well-being in South Africa, and the linkages between human well-being and ecosystem services. However, it is challenging to construct a well-being bundle that is made up of indicators which do not bias the analysis towards modern aspirations and definitions of human well-being. It could be argued that indicators like income, education and unemployment may be important measures of well-being in wealthy modern, often urban societies (red loop systems), but are less important at determining levels of well-being in green-loop systems, i.e. rural, close-knit communities where not all interactions are cash-based and other aspects of well-being may be more highly valued. As our results show, simply identifying areas in which traditional red-loop well-being indicators exhibit low values is not necessarily sufficient to detect green-loop systems. Our approach is reliant on existing, publicly available data that are not tailor-made to measure all aspects of human well-being, which may partly explain why our well-being bundle was not entirely effective in identifying green-loop systems. A modified human well-being bundle that includes indicators more suited to represent green-loop communities (indicators like community cohesion and identity, resource tenure, or access to nature, for example) might help differentiate the green-loop/red-loop social-ecological systems more successfully than the bundle chosen here. However, the challenge of finding data at relevant (sub-national) scales for these more unorthodox well-being measures remains difficult to overcome [65].

Furthermore, we examined human well-being through objective measures only, without taking into account subjective well-being or self-reported happiness. Research on subjective well-being is extensive and growing [66–69], but due to data limitations at the scale of our analysis we chose to focus on objective well-being in South Africa. However, some of the objective indicators chosen for this study (level of income, education and unemployment) have been shown to be highly correlated with subjective well-being, both in developing countries generally [70, 71], and within South Africa more specifically [72, 73]. The low income bundle areas in this study are therefore likely to be characterised not only by an overall low level of objective well-being, but also by low levels of subjective well-being. It is more difficult to deduce the level of subjective well-being in the other two bundle types, since they each present a mix of factors that have been shown to contribute to subjective well-being, i.e. the high income bundle has high levels of income and education but also high levels of unemployment, while the medium income bundle has low unemployment but also low education levels. In further research it would be pertinent to include subjective well-being indicators when considering the links between human well-being and ecosystem services, as there is some evidence for a positive relationship between ecosystem services and life satisfaction at the country level [74]. A case study at local scale where both objective and subjective measures of well-being are considered with respect to ecosystem service use would greatly further this line of research.

In general, we acknowledge that the scale of our analysis (at the municipal level) excludes a finer analysis of more localized and disaggregated interactions between well-being and ecosystem services, which are the focus of much of the research done in this field (e.g. [75–78]). Especially in South Africa, where inequality is high, variability in the human well-being indicators *within* each of the municipalities is likely to be high as well. More fine-scale data at the sub-municipal level would help to identify particularly unequal and diverse municipalities in terms of well-being and resource use dynamics, but is unavailable at present for all indicators assessed here. Further research is also required to investigate what drives the differentiation into green-loop, transition and red-loop systems within low income bundle areas, and could include an analysis of variables such as population density, land tenure, cultural identity, or land degradation.

## Conclusions

The human well-being bundles identified in this study represent a novel approach to mapping well-being, and illustrate that well-being across South Africa is differentiated and nuanced in a way that may be missed by single well-being indicators or composite indices that mask diversity between well-being constituents. Furthermore, this approach allowed a spatially explicit comparison of human well-being and ecosystem service use patterns at the sub-national scale, and we found that human well-being bundles could partly identify the green-loop, transition and red-loop systems that were previously identified using ecosystem service use bundles: Almost all medium and high income bundle areas coincided with low ecosystem service use (red-loop systems), and the majority of low income bundle areas coincided with medium and high ecosystem service use (transition and green-loop systems). We hypothesize that the remaining discrepancies in the well-being and resource use patterns may be due to time lags between fast-changing ecosystem service use dynamics and slower-changing well-being variables. Such asynchronous dynamics are to be expected in social-ecological systems [79], but are not yet sufficiently acknowledged and accounted for in ecosystem service studies that aim to establish a connection between ecosystem services and human well-being. We suggest that this approach of using human well-being bundles to explore green-loop, transition and red-loop systems and their ecosystem service use can be usefully translated to other parts of the world, since it is based on existing socio-economic data that most countries collect regularly and at sub-national levels. Countries may use this approach to assess potential links between human well-being and ecosystem service use, initially to identify areas of concern (e.g. where well-being is low and land degradation is high), and then to focus more detailed investigations in those areas.

## Supporting Information

S1 Fig Correlations between all ecosystem service (ES) use and human well-being (HWB) indicators. Numbers in the boxes represent Spearman’s ρ (r_S_); only coloured boxes represent significant (p < 0.05) correlations between indicators (blue = significantly positive correlations, red = significantly negative correlations).
(PDF)

S2 Fig Multidimensional scaling ordination diagram for all South African municipalities (n = 234), based on five human well-being measures. Colours indicate the three different human well-being bundle types identified in the cluster analysis.
(PDF)

S3 Fig Human well-being bundles when k-means clustering was restricted to two clusters. (PDF)

S4 Fig Human well-being clusters resulting from a hierarchical cluster analysis. (PDF)
