# Supplementary material for: An Exploration of Human Well-Being Bundles as Identifiers of Ecosystem Service Use Patterns
Source: PLoS One. 2016 Oct 3;11(10):e0163476. doi: 10.1371/journal.pone.0163476 (PMC5047452; doi:10.1371/journal.pone.0163476)
Supplement: S1 Fig — Numbers in the boxes represent Spearman’s ρ (rS); only coloured boxes represent significant (p < 0.05) correlations between indicators (blue = significantly positive correlations, red = significantly negative correlations). (PDF) [file pone.0163476.s001.pdf]

S1 Appendix

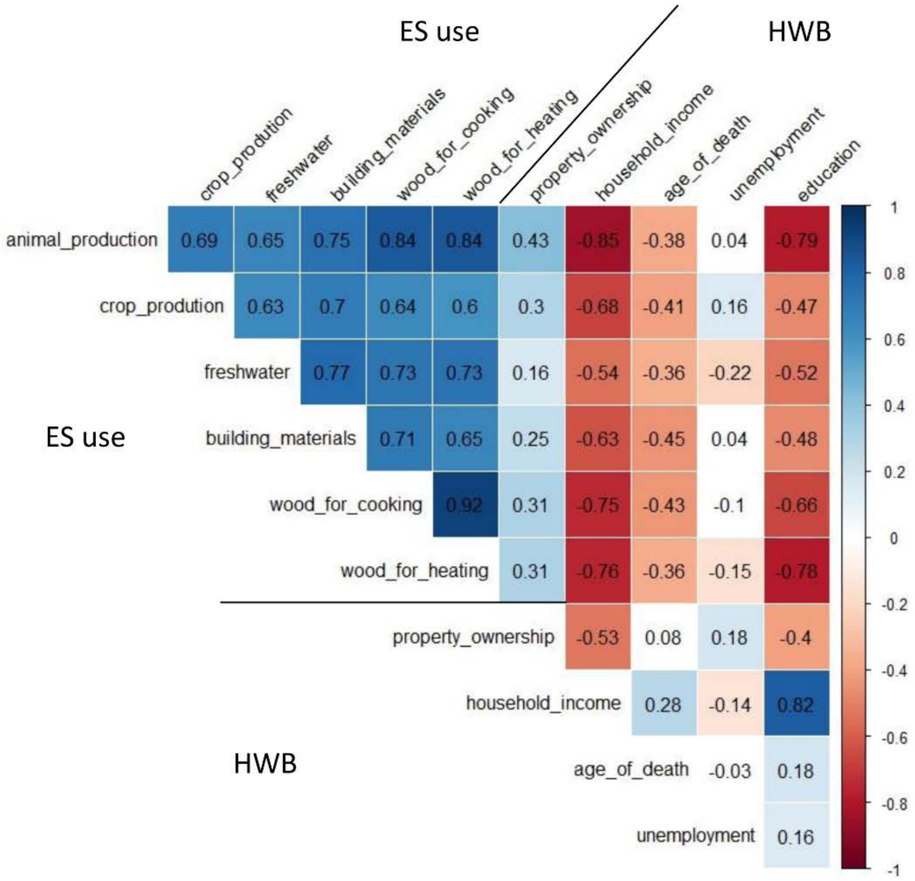

S1 Fig. Correlations between all ecosystem service (ES) use and human well-being (HWB) indicators. Numbers in the boxes represent Spearman's ρ (r_S); only coloured boxes represent significant ($p < 0.05$) correlations between indicators (blue = significantly positive correlations, red = significantly negative correlations).
