# Supplementary material for: An Exploration of Human Well-Being Bundles as Identifiers of Ecosystem Service Use Patterns
Source: PLoS One. 2016 Oct 3;11(10):e0163476. doi: 10.1371/journal.pone.0163476 (PMC5047452; doi:10.1371/journal.pone.0163476)
Supplement: S2 Fig — Colours indicate the three different human well-being bundle types identified in the cluster analysis. (PDF) [file pone.0163476.s002.pdf]

S2 Appendix

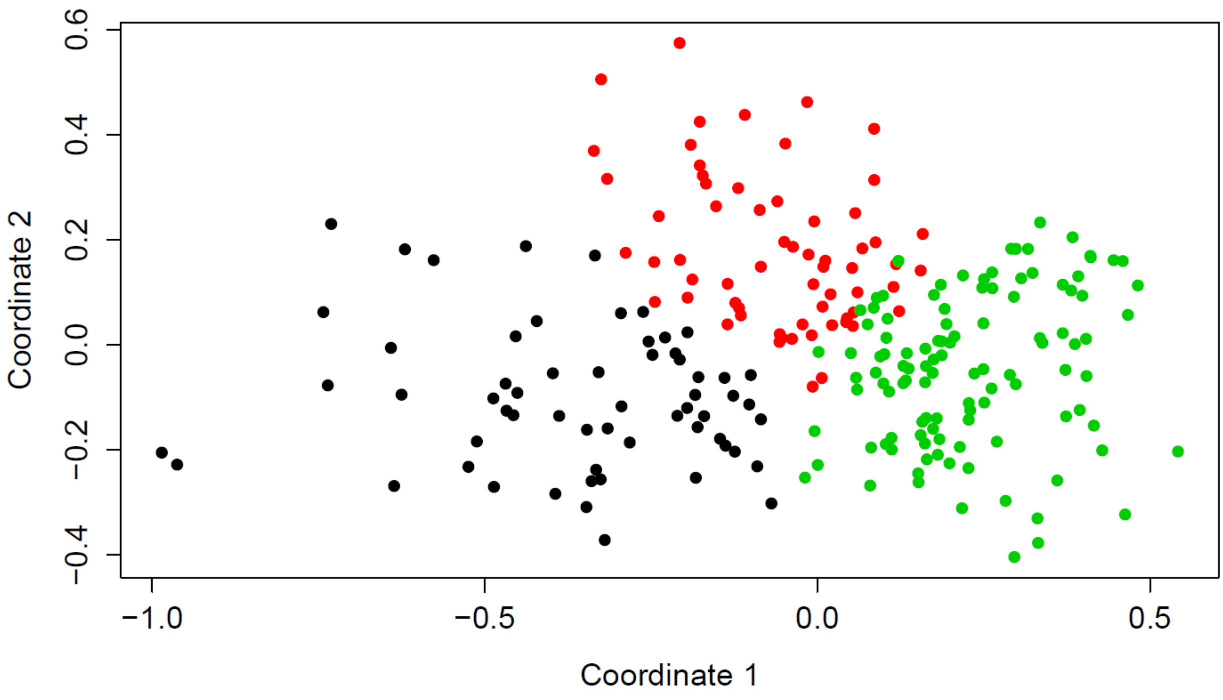

S2 Fig. Multidimensional scaling ordination diagram for all South African municipalities (n = 234), based on five human well-being measures. Colours indicate the three different human well-being bundle types identified in the cluster analysis.
