# Supplementary material for: An Exploration of Human Well-Being Bundles as Identifiers of Ecosystem Service Use Patterns
Source: PLoS One. 2016 Oct 3;11(10):e0163476. doi: 10.1371/journal.pone.0163476 (PMC5047452; doi:10.1371/journal.pone.0163476)
Supplement: S3 Fig — (PDF) [file pone.0163476.s003.pdf]

S3 Appendix

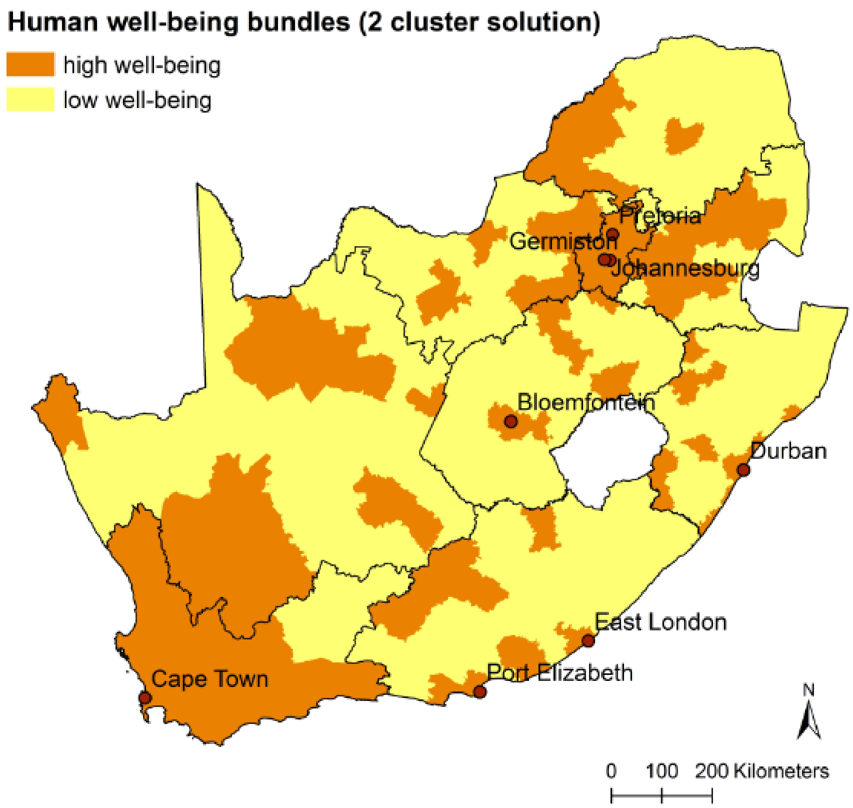

S3 Fig. Human well-being bundles when k-means clustering was restricted to two clusters.
