# Supplementary figures and images for: An Exploration of Human Well-Being Bundles as Identifiers of Ecosystem Service Use Patterns
Source: PLoS One. 2016 Oct 3;11(10):e0163476. doi: 10.1371/journal.pone.0163476 (PMC5047452; doi:10.1371/journal.pone.0163476)

## S4 Appendix

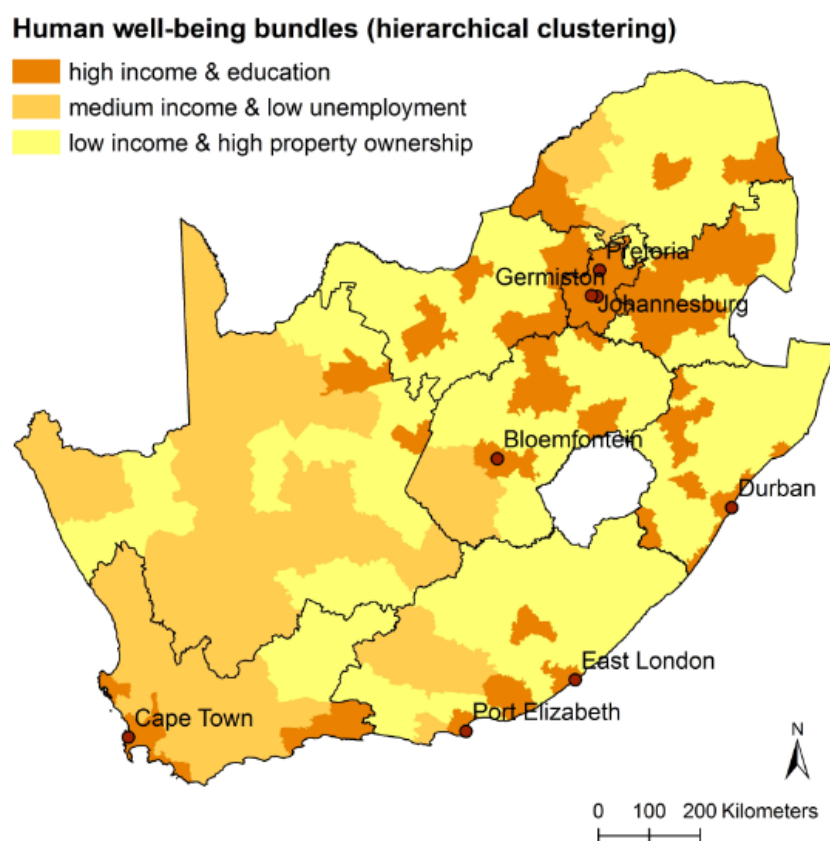

**S4 Fig. Human well-being clusters resulting from a hierarchical cluster analysis.**

Supplement: S4 Fig — (PDF) [file pone.0163476.s004.pdf]
